# Systematic Review on In Situ Laser Fenestrated Repair for the Endovascular Management of Aortic Arch Pathologies

**DOI:** 10.3390/jcm12072496

**Published:** 2023-03-25

**Authors:** Thomas Le Houérou, Petroula Nana, Mathieu Pernot, Julien Guihaire, Antoine Gaudin, Erol Lerisson, Alessandro Costanzo, Dominique Fabre, Stephan Haulon

**Affiliations:** 1 Aortic Center, Marie Lannelongue Hospital, Groupe Hospitalier Paris Saint Joseph, Paris Saclay University, Av. de la Resistance, 92350 Le Plessis-Robinson, France; 2 UMCV, Hôpital Cardiologique de Haut-Leveque, Bordeaux University Hospital, 33000 Bordeaux, France

**Keywords:** in situ, laser, FTEVAR, aortic arch, technical success, mortality, stroke

## Abstract

Background: In situ laser-fenestrated thoracic aortic endovascular repair (FTEVAR) has emerged as a valuable alternative for aortic arch management. This review assessed the early and follow-up outcomes of in situ laser-FTEVAR in aortic arch pathologies. Methods: The PRISMA statement was followed. The English literature was searched, via Ovid, until 15 October 2022. Observational studies, published after 2000, reporting on early and follow-up outcomes for the in situ laser-FTEVAR were eligible. The Newcastle–Ottawa Scale was used to assess the risk of bias. Primary outcomes were the technical success, stroke, and mortality at 30-days, and the secondary were the mortality and reintervention during follow-up. Results: Six retrospective studies from 591 and 247 patients were included. Fifty-nine (23.9%) patients were managed for aortic arch aneurysms and 146 (59.1%) for dissections; 22.6% of them for type A. Technical success was at 98% (range 90–100%). Eight patients died (3.2%) and 11 cases presented any type of stroke (4.5%) during the 30-day follow-up. The mean follow-up was 15 months (1–40 months). Ten deaths were reported (4.2%); one was aortic-related (10%). Thirteen re-interventions (6.0%) were performed. Conclusions: In situ laser-FTEVAR for aortic arch repair may be performed with high technical success and low 30-day and midterm follow-up mortality, stroke, and re-intervention rates when applied in well selected patients and performed by experienced teams.

## 1. Introduction

Current recommendations suggest the use of endovascular repair in patients suffering from aortic arch and thoracic aorta pathologies and are considered unfit for open repair [1]. Recent data has shown that fenestrated or branched endovascular repair (F/BTEVAR) may be a valuable solution for the endovascular management of aortic arch diseases, with encouraging initial outcomes in terms of stroke and mortality [2,3,4]. However, the need for re-intervention is still an issue [5]. In addition, the application of F/BTEVAR using customized devices is restricted by specific anatomic criteria, while the limited availability of the devices may hamper their use in urgent and emergent cases, confining the target population that could benefit from an endovascular arch repair [2]. Off-the-shelf techniques including surgeon modified endografts and parallel grafts have been applied to address these issues [2,6,7].

Among them, in situ fenestration of standard endografts (in situ FTEVAR) using laser or mechanical means has been described and is used to extend the endovascular management of the arch on proximal landing zones [2,8]. Recent published data have shown that in situ FTEVAR has been associated with high technical success and acceptable early mortality and stroke rates [8]. The use of standard devices in this setting are out of the instructions for use from the manufacturers while various complications including fabric damage need to be evaluated [8,9,10]. Long-term data regarding the associated mortality and re-intervention rates are lacking, raising questions on the durability of the technique [2,3,4].

Along these lines, the aim of this systematic review was to assess the technical success, stroke, and mortality rates at 30 days as well as the mortality and re-intervention rates during the available follow-up in patients managed with in situ laser FTEVAR for aortic pathologies affecting the arch.

## 2. Materials and Methods

### 2.1. Eligible Studies

**Figure 1:**
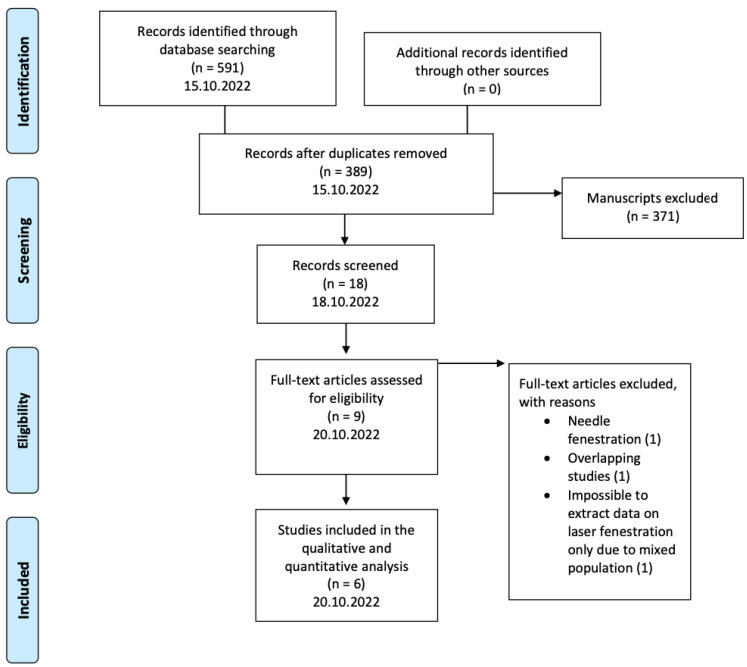
The flowchart of the selection process according to the PRISMA statement. Six studies were finally included in this systematic review.

The Preferred Reporting Items for Systematic Reviews and Meta-Analyses (PRISMA) guidelines were followed (Figure 1) to conduct the current review [10]. Retrospective and prospective observational studies and case series of the English medical literature, published after 2000, reporting on in situ laser FTEVAR outcomes for aortic arch repair were considered eligible. Technical success, stroke, and mortality rates at 30 days, and mortality and re-intervention rates during the available follow-up should be reported. Studies reporting on endovascular management of the arch with other techniques than in situ laser FTEVAR, such as F/BTEVAR with custom-made devices, surgeon modified TEVAR with mechanical creation of fenestrations, or TEVAR using the parallel graft technique were excluded. Furthermore, case reports and case series with less than five patients were considered ineligible. Among the studies presenting potential overlap, only the most recent data were included in this analysis.

### 2.2. Ethical Considerations and Approval

Scientific Council approval and patient consent were not required due to the nature of the analysis.

### 2.3. Search Strategy

A systematic search in MEDLINE and EMBASE via the Ovid and CENTRAL databases of the English medical literature was performed according to the PICO [Patient; Intervention; Comparison; Outcome] model (Supplementary Table S1) [11,12]. The endpoint was set for 15 October 2022 The following search items including Expanding Medical Subject Heading (MeSH terms) were used in various combinations: aortic arch, in situ, laser, fenestration, FTEVAR, mortality, morbidity, and re-intervention. The reference lists of the included full texts were assessed for any additional eligible studies. A consequent scrutiny was performed after full-text assessment by two independent authors (P.N. and M.P.). Any discrepancy during the study selection process was resolved after discussion with a third author (S.H.).

### 2.4. Data Extraction

A standardized Microsoft Excel file was generated and the extracted data included the studies’ characteristics (author, journal, date of publication, study design, timespan, country/center/database, aim), baseline demographics (age, sex), indication to treat, setting; urgent or elective, landing zone according to Ishimaru’s criteria, number and type of target vessels (TVs), devices (main endograft device, laser characteristics, and bridging stents), and intra-operative details (duration of operation and fluoroscopy time). Technical success, and its definition when available as well as mortality, morbidity [spinal cord ischemia (SCI), stroke; minor or major, retrograde dissections or new stent induced dissections, acute kidney injury (AKI), cardiac and pulmonary adverse events] and endoleaks at 30 days were recorded. Mortality (total and aorta-related), TV patency, retrograde dissections, endoleaks, and re-interventions during the available follow-up were extracted when available. Data extraction was performed by two independent authors (P.N. and M.P) and in the case of discrepancy, a third author was advised (S.H.).

### 2.5. Quality Assessment

The quality assessment of the studies was performed using the Newcastle–Ottawa Scale (NOS) [13]. NOS evaluates three methodological domains: selection, comparability of cohorts on the design or analysis, and the assessment of outcomes. A star system, with a maximum of nine stars, was used and studies with at least seven stars were considered of high quality [13]. The assessment was performed by two independent authors (P.N. and M.P.) and any discrepancy was resolved after advice from a third author (S.H.).

### 2.6. Outcomes

The primary outcomes of this systematic review were technical success, stroke, and mortality rates at 30 days in patients managed with in situ laser FTEVAR for aortic arch pathologies. Secondary outcomes included mortality and re-intervention rates during the available follow-up.

### 2.7. Statistical Analysis

Descriptive data are mainly presented since this systematic review was not comparative. Continuous data were reported as the mean ± standard deviation. Categorical data were expressed as absolute numbers with the associated range. The effect of measures for technical success, early and follow-up mortality, cerebrovascular events, and endoleaks were presented as percentages or proportions of the included studies for each outcome. For the missing data, there was no imputation and the effect of measure of each outcome was estimated on the cohort of the studies reporting on each specific outcome. Statistical analyses used SPSS 20.0 software (IBM Corp, Armonk, NY, USA).

## 3. Results

The initial search yielded 591 manuscripts. The search strategy is presented in Supplementary Table S2. After deduplication and article exclusion, six studies were included in this review (Figure 1) [14,15,16,17,18,19]. All of them were of a retrospective nature [14,15,16,17,18,19]. The main characteristics of the studies are presented in Table 1.

### 3.1. Patient Cohort

In total, 247 patients were included; 173 were males (70%) with a mean age at 63 years (range 37–82 years) [14,15,16,17,18,19]. Different aortic arch pathologies were managed including 59 (23.9%) cases of aortic arch aneurysms and 146 (59.1%) of acute or subacute dissections; 33 (22.6%) of them were type A [14,15,16,17,18,19]. Among the aneurysms, 16 (27.1%) were post-dissection. The urgency of the repair was reported in four studies; 199 (80.6%) cases were considered urgent or emergent [14,15,18,19]. Regarding the anatomic criteria for in situ laser FTEVAR application, coronary artery or aortic valve involvement, proximal landing less than 15–20 mm, proximal diameter ≥40 mm, extension of the disease to the supra-aortic trunks (dissected target vessels or aneurysmal orifices), and target vessel steep take off and tortuosity were considered and reported as exclusion criteria for in situ laser FTEVAR [15,17,19].

A variety of main endografts and laser devices were used, as reported in Table 2. All studies reported the number and type of target vessels as well as the proximal landing zones [14,15,16,17,18,19]. In total, 321 TVs were revascularized using laser fenestration: among them, 41 innominate arteries, 52 left common carotid arteries, 224 (69.8%) left subclavian arteries, and four aberrant subclavian arteries. Regarding the landing zones according to Ishimaru’s criteria, in 47 (19%) cases, the main device was deployed to Ishimaru’s zone 0, in 38 in zone 1, and in 162 (65.6%) in zone 2. Five studies reported the specific bridging stent type including covered self and balloon expandable stents [14,16,17,18,19]. The remaining study referred to the use of both bare metal and covered bridging stents without further detail [15]. Three studies reported on the use of adjacent procedures for TV revascularization including one back-table fenestration for the preservation of an innominate artery [14], three chimneys for the preservation of the left common carotid artery [16], and 12 extra-anatomic bypasses [19].

### 3.2. Intra-Operative Details

Four studies reported the use of both percutaneous and cut down for the access vessels’ approach [14,15,17,18]. The mean operation time was 200 min (range 40–350 min) [14,17,19]. The fluoroscopy time and dose area product (DAP) were not reported in any study.

Technical success definition was reported in three studies [15,17,18]. Liu et al. defined technical success as the accomplishment of aortic endograft and TV stent placement [15]. Yan et al. reported as technical success the successful three-branch fenestration, or successful brachiocephalic trunk and left carotid artery fenestration, when the right vertebral artery was considered dominant [17] and Li et al. as the successful laser fenestration of the stent graft fabric, wire cannulation, balloon dilation of fenestration, and deployment of the TV covered stent without type I or IIIC endoleaks detected in the completion angiography [18]. Technical success was reported in all studies, and it was estimated at 98% (range 90–100%) [14,15,16,17,18,19]. No intra-operative endoleaks or deaths were reported.

### 3.3. 30-Day Outcomes

Early outcomes are described in Table 3. Eight patients died during the early follow-up (3.2%, 8/247) [14,15,16,17,18,19]. In three studies, early mortality events were described including one aortobronchial fistula, one stent-induced dissection followed by aortic rupture, and one abdominal aortic rupture (3/6, 50%) [14,17,18]. Eleven patients presented any type of stroke (4.5%, 11/247), one was considered major while the remaining were described as minor strokes or transient ischemic attacks [14,15,16,17,18,19]. Three SCI events were reported in three studies, leading to a 1.2% rate [14,18,19]. Two retrograde dissections were recorded during the 30-day follow-up in two studies [14,18]. Three early re-interventions were reported in two studies, all due to access complications [14,15].

### 3.4. Follow-Up Outcomes

The mean follow-up was 15 months, ranging from 1 to 40 months (Table 4) [14,15,16,17,18,19]. Ten additional deaths were recorded (4.2%); one was aortic-related (10%, 1/10) [14,15,16,17,18,19]. No further strokes or SCI events were detected. TV patency was reported in four studies and was 100%, while one event of retrograde dissection was recorded during follow-up [14,15,16,18]. Twenty endoleaks were stated in all studies (8.3%, 20/239; four were type I (2 type Ia, 1 type Ib, and 1 type Ic) and nine were type III [14,15,16,17,18,19]. In total, 13 re-interventions (6.0%, 13/216) were performed [14,16,17,18,19]. The specific indications for re-intervention during follow-up are presented in Table 4. Most reinterventions were attributed to endoleak management.

### 3.5. Risk of Bias

According to the NOS, only one study received seven stars and was considered as having a low risk of bias [18]. All of the remaining studies were characterized as poor quality (Table 5) [14,15,16,17,19]. All studies were retrospective, and a variety of confounders was detected including the small number of cases, differentiation of technical details, surgeon and patient selection, limited follow-up, and no report of loss to follow-up.

## 4. Discussion

### 4.1. Findings in the Current Literature

In situ laser fenestration represents a valuable off-the-shelf solution for aortic arch endovascular repair in patients considered as high-risk for conventional open management and present anatomic characteristics that do not permit the application of custom-made solutions [1,18,19]. Despite the retrospective design and limited number of studies and patients, this review showed that in situ laser fenestration for the management of aortic arch diseases represents a feasible and reliable treatment option that is associated with a high technical success of 98% and acceptable complication and mortality rates in the short- and mid-term follow-up when applied in well-selected patients and performed by experienced hands.

According to the latest available expert consensus, in situ fenestration, using mechanical means or laser, represents an alternative endovascular option in cases needing an extension of coverage to a more proximal landing zone [1,20,21]. However, as in situ fenestration is an off-label procedure, it would be better performed as an emergent bailout technique due to the potential damage to endograft fabric and the associated risk of endoleaks [22]. In this analysis, the endoleak rate was estimated at 8% while type III endoleaks represented 3.5%, without further definition of whether these endoleaks were related to fabric tears or disconnection of the modules [18]. Supra-aortic vessel anatomy is an important factor that can affect the successful creation of fenestration, while the bridging stent choice could also play a significant role in the prevention of endoleaks, as described analytically later in the technical note of this article. The current data on self-expanding stents show that they may be related to type III endoleaks, which set the indication for further intervention [18]. The use of balloon-expandable stents could be a safer option to reduce the risk of type III endoleaks.

While custom-made branched or fenestrated devices set specific instructions for use including a landing zone of more than 40 mm and a diameter of landing zone less than 38 mm, off-the-shelf solutions such as laser fenestration can overcome these restrictions and expand the targeted patient population that would benefit from an endovascular approach [23,24]. Currently available standard thoracic devices providing diameters up to 46 mm, and tapered morphologies permit adaption to many different anatomies while the familiarity and long experience of physicians with standard devices permit endograft selection, adapting better to the patients’ needs and anatomy [25,26]. However, in our opinion, the restriction of the landing zone length should be respected in any device applied as the aortic arch represents a segment where the anatomy constrains differently due to the curvature and higher hemodynamic pressures.

Regarding the post-operative outcomes, it should be noted that paraplegia was rare, as previously reported in patients managed for arch disease using endovascular means [19,27]. Further analyses based on larger patient samples could shed some more light on the association of laser fenestration and neurological events. Data arising mainly from fenestrated and branched arch repair showed a stroke risk rate up to 15% earlier in the literature, while increasing experience achieved a decrease in the potential cerebrovascular complication rate [28,29]. In this analysis, the stroke rate was 5% and within the reported limits in the literature for endovascular arch repair, while recent data on hybrid repair, using SAT debranching, reported a stroke rate up to 14% [18,28,29,30,31]. Anatomic factors, as shaggy or heavily calcified aorta as well as the role of medical treatment using single or double antiplatelet therapy, could not be investigated in the current analysis, despite their potentially significant role in stroke prevention, and need further assessment.

Due to the encouraging outcomes of laser fenestration for the revascularization of the LSA, more extended arch repairs have been reported, with double or triple SAT fenestrations. Thus, more complex aortic arch pathologies could be managed using a less invasive endovascular approach [32]. However, the realization of an extended treatment of the aortic arch with in situ laser fenestration requires the implementation of atemporary extracorporeal cerebral perfusion during supra-aortic trunk coverage, until the fenestrations are performed [32]. Temporary extracorporeal circulation or adjacent shunts, connected within the different sheaths, have been described to secure cerebral perfusion.

In 2020, Qin et al. published their experience on the management of patients with type A aortic dissection, contraindicated for conventional surgical management [33]. The two-year survival rate was estimated at 77%; very positive outcomes, especially when considering the poor prognosis of type A dissection medical management [33]. Laser fenestration can provide a therapeutic option in the context of the emergency management of complex aortic pathology in patients considered unfit for any other surgical or interventional repair [34]. In this review, the specific anatomical characteristics of type A dissection treated with in situ laser fenestration were lacking while type A aortic dissection repair was mainly performed, as a second stage procedure, in the distal part of the ascending aorta, with a proximal sealing in a “non-diseased” area.

### 4.2. Technical Aspects

In situ fenestration for the treatment of aortic arch pathologies is mostly performed in urgent or semi-urgent cases in the setting of acute aortic syndromes including complicated type B dissections, penetrating ulcers and intramural hematomas, isthmic ruptures, or symptomatic aneurysms of the distal aortic arch. The first case of retrograde mechanical fenestration of the subclavian artery dates was published in 2004 [35], and the first laser fenestration was reported in 2009 by Murphy et al. [36]. The technique was initially described using a left humeral access with a 7Fr introducer via a retrograde approach. Fenestrations of one or more supra-aortic trunks using an anterograde approach was also described [36].

To minimize the duration of ischemia, open surgical or endovascular access of the brachial, axillary, or carotid artery is performed before aortic stent-graft deployment. When sealing can be achieved only with bridging stent diameters larger than 10 mm, axillary access is recommended (open or percutaneous approach) [37].

The use of long sheaths is mandatory to obtain adequate support. The use of pre-curved sheaths can also be helpful in vessels with significant tortuosity. A reduced angle between the aorta and the target vessel makes fenestration creation more challenging. Thus, type 1 and 2 aortic arches have more favorable anatomies compared to type 3 when in situ fenestrations are considered.

**Figure 2:**
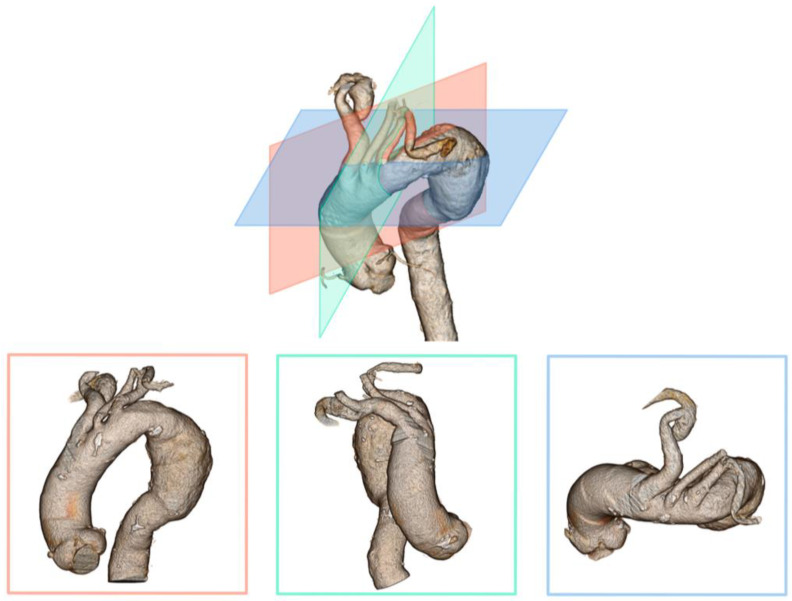
Three-dimension reconstruction of the aortic arch and the associated dimension graphic depicting the significance of the target vessel angle to the aortic wall in three planes.

**Figure 3:**
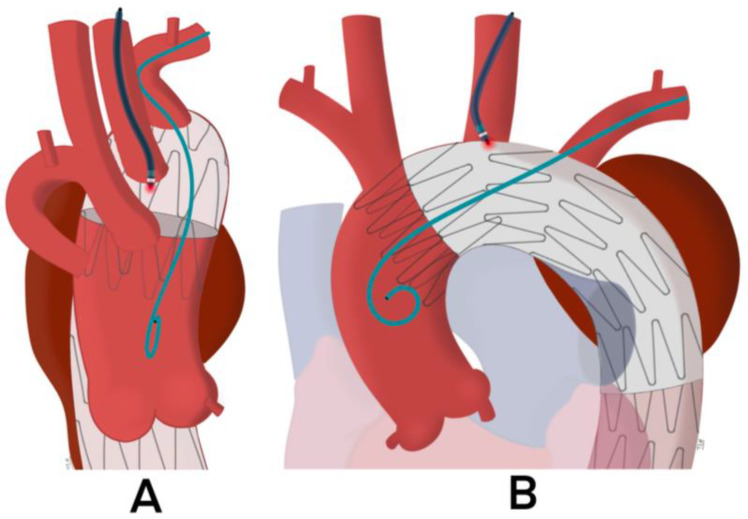
After the apposition of the guide catheter in contact with the aortic graft, the appropriate positioning should be evaluated in the anteroposterior and lateral view before the laser probe activation. The distal end of the guide catheter must be positioned at a 90° angle with the surface of the fabric of the stent graft. ((**A**): lateral and (**B**): anteroposterior view): XXXXXXXXXX.

To compensate for the angulation between the greater curvature and the target vessel, an additional 6 or 7Fr JR4 or IM guide-catheter is recommended to facilitate the orientation of the coaxial system to the surface of the aortic graft. The use of a steerable catheter can also be considered. Once the guide-catheter is in contact with the fabric of the endograft, it is essential to verify its correct positioning. This is an essential step before activating the laser probe. The distal end of the guide-catheter must be positioned at the top of the “dome” of the TEVAR, pointing to the aortic center lumen. For this purpose, it is recommended to check its position on two orthogonal planes intersecting at the ostium of the target artery (Figure 2 and Figure 3). If there is too much angulation between the target artery and the greater aortic curvature, there is a risk of failed fenestration. In this setting, the laser probe often slips between the TEVAR and the anterior aortic wall.

**Figure 4:**
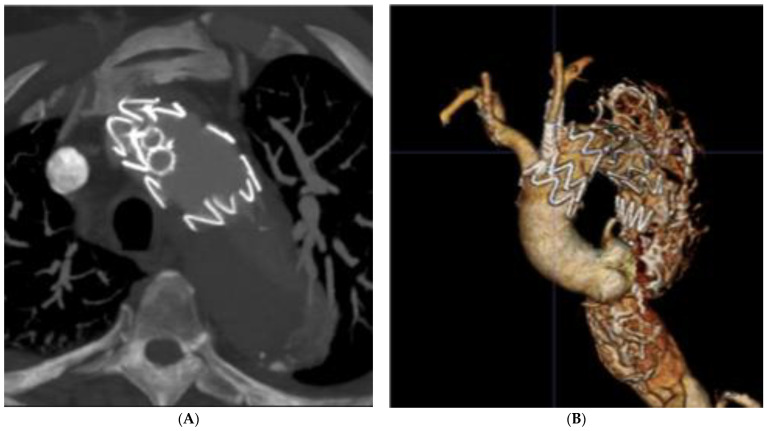
Post-operative CT scan 3D volume rendering (**A**) of the in situ laser fenestrations of the left common carotid and left subclavian arteries. The 3D reconstruction (**B**) shows fenestrations position and patency.

Concerning the choice of the laser probe, previous studies have reported the use of the Spectranetics Turbo-Elite coronary atherectomy probe, which exists in several diameters from 0.9 to 2.5 mm, compatible with 0.014″ to 0.035″ guidewires. Progressive dilatation with coronary and non-compliant balloons is necessary to enlarge the fenestration while the use of cutting balloons can facilitate the passage of larger diameter balloons. Finally, balloon-expandable covered stents are better adapted to provide adequate sealing at the level of the fabric tear and into the target vessel (Figure 4).

### 4.3. Limitations

This systematic review included data across six retrospective studies to evaluate the associated technical success, stroke, mortality, and re-intervention rates in patients managed with in situ laser FTEVAR for different aortic arch pathologies. The retrospective nature and additional confounders introduced significant bias that should be acknowledged. The risk of bias among the included studies varied considerably. Only one study was considered of high quality according to the evaluation by NOS. As the published experience is limited (247 cases), the findings of this analysis may not depict real world data but rather the outcomes of experienced centers. In addition, the study by Li et al. included more than half of the patients of the current analysis and mainly drove the findings [18]. Furthermore, technical details such as patient selection criteria and anatomic characteristics were not available in most studies. Urgent and elective cases were included, adding further potential bias. Regarding specific definitions, only half of the studies reported a definition of technical success. Long-term data were missing from all studies. None of the studies reported loss to follow-up. Further analyses providing larger patient samples and extended follow-up are needed.

## 5. Conclusions

According to the available limited literature, in situ laser FTEVAR for aortic arch repair may be performed with high technical success and low 30-day stroke and mortality rates. Mortality and re-intervention rates were low during the 15-month follow-up. Both patient selection and procedure performance in experienced centers should be acknowledged. Further analyses are needed to provide firm conclusions on the safety, efficacy, and durability of the in situ laser FTEVAR for aortic arch repair.

## Figures and Tables

**Table 1:** The main characteristics of the included studies and indications to treat.

Author	Year	Country	Type of Study	Timespan	No. of Patients	Age	Aortic Arch Aneurysm	Dissections Involving the Aortic Arch	IMH or PAU
Redlinger et al. [14]	2013	USA	Retrospective, single center	2009–2012	22	57 (37–83)	12 (8 chronic dissections)		6
Liu et al. [15]	2018	China	Retrospective, single center	2014–2017	23	66 ± 10.8 (41–82)	7		4
Sonesson et al. [16]	2019	Sweden	Retrospective, single center	2014–2016	10	68	7	2	1
Yan et al. [17]	2020	China	Retrospective, single center	2016–2018	20	67		20 (20 type A)	
Li et al. [18]	2020	China	Retrospective of prospective data, single center	2017–2019	148	54.9 ± 12.9	17	120 (13 type A)	11
Evans et al. [19]	2021	USA	Retrospective, single center	2017–2020	24	62 (24–82)	16 (8 chronic dissections)	4	

Footnotes: IMH: intramural hematoma; PAU: penetrating aortic ulcer.

**Table 2:** Technical characteristics of the devices used for in situ laser FTEVAR including the main endografts and bridging stents.

Author	Type of Laser	Main Graft	3 Fenestrations	2 Fenestrations	1 Fenestration	Bridging Stents
Redlinger et al. [14]	2.0- to 2.5-mm Turbo Elite laser catheter, Spectranetics, Colorado Springs, CO, USA	Talent or Valiant (Medtronic, Santa Rosa, CA, USA), Zenith TX2 (Cook Inc., Bloomington, IN, USA)	0	0	22	iCAST (Atrium, Hudson, NH, USA)
Liu et al. [15]	810 nm wavelength laser, Gigaa Optronics Technology Co. Ltd., Wuhan, China, application of 18 W for 3–5 s	TAG/cTAG (W.L. Gore & Associates, Flagstaff, AZ, USA), Valiant (Medtronic, Santa Rosa, CA, USA), Ankura (Lifetech, Shenzhen, China)	2	3	6	Covered and bare metal stents
Sonesson et al. [16]	308 nm wavelength laser, 0.9 mm Turbo Elite over the wire, Spectranetics, Colorado Springs, CO, USA	Zenith Alpha (Cook Inc., Bloomington, IN, USA)	0	0	10	Begraft (Bentley, Hechingen, Germany), Fluency (Bard Peripheral Vascular, Tempe, AZ, USA), Visipro (Medtronic, Dublin, Ireland), Advanta (Atrium Europe B.V, Mijdrecht, the Netherlands), Lifestream (Bard Peripheral Vascular, Tempe, AZ, USA), Protégé (ev3 Endovascular Inc., Plymouth, MN, USA)
Yan et al. [17]	810 nm wavelength diode laser, Eufoton Company, Trieste, Italy, application of 18 W for 3 s	cTAG (W.L. Gore & Associates, Flagstaff, AZ, USA)	18	2	0	Fluency (Bard Peripheral Vascular, Tempe, AZ, USA) for LCCA, Endurant limb if TBA >13 mm (Medtronic, Santa Rosa, CA, USA)
Li et al. [18]	980 nm wavelength laser VELAS, GIGAA Laser, Wuhan, China, application of 18 W for 3 s	90 Valiant (Medtroni, Santa Rosa, CA, USA), 30 cTAG (W.L. Gore & Associates, Flagstaff, AZ, USA), 19 Zenith (Cook Inc., Bloomington, IN, USA), 6 Ankura (Lifetech, Shenzhen, China), 2 Hercules (Microport, Shanghai, China), 1 E-vita Thoracic 3 g (Jotec, Hechingen, Germany)	11	13	124	119 Fluency (Bard Peripheral Vascular Tempe, AZ, USA), 58 Viabahn (W.L. Gore & Associates, Flagstaff, AZ, USA), 2 Endurant limbs (Medtronic, Santa Rosa, CA, USA)
Evans et al. [19]	308 nm wavelength, 2.3 mm 0.035 Spectranetics laser (Philips, Amsterdam, Netherlands), with a fluency setting of 40 mJ/mm^2^ and repetition rate of 60 Hz	cTAG (W.L. Gore & Associates, Flagstaff, AZ, USA), Valiant (Medtronic, Santa Rosa, CA, USA)	0	6	18	VBX (W.L. Gore & Associates, Flagstaff, AZ, USA)

**Table 3:** Early outcomes including mortality, morbidity, and re-interventions in patients managed with in situ laser FTEVAR.

Author	Early Outcomes	Technical Success	Mortality	Stroke	Spinal Cord Ischemia	Re-Interventions	Acute Kidney Injury
Redlinger et al. [14]	30-day	100%	1	0	1	NA	0
Liu et al. [15]	30-day	100%	0	1	NA	1	NA
Sonesson et al. [16]	30-day	90%	0	1	NA	NA	NA
Yan et al. [17]	30-day	100%	2	1	NA	NA	NA
Li et al. [18]	30-day	97.8%	3	5	0	NA	NA
Evans et al. [19]	30-day	100%	2	3	2	NA	4

Footnotes: NA: non-applicable.

**Table 4:** Follow-up data including mortality and re-intervention rates in patients managed with in situ laser FTEVAR.

Author	Follow-Up Duration	Mortality	Aorta-Related Death	TV Patency	Re-Interventions	Endoleak	Reasons for Re-Intervention
Redlinger et al. [14]	11 (1–40)	2	0	100%	2	2	Coils for ET II from LSA
Liu et al. [15]	10.5 ± 5.7	0	0	100%	NA	0	
Sonesson et al. [16]	27	2	1	100%	1	2	Type Ic from LSA
Yan et al. [17]	16 (3–26)	1	NA	NA	0	3	
Li et al. [18]	15 ± 5	2	0	100%	1	7	Type Ib endoleak, extension LSA
Evans et al. [19]	9 (1–29)	3	0		9	6	6 embolization and extension for ET III, 1 angioplasty for bypass stenosis, 1 bypass for endoleak, 1 hematoma

Footnotes: ET: endoleak type; LSA: left subclavian artery; NA: non-applicable; TV: target vessel.

**Table 5:** Assessment of quality reporting in the gathered literature according to the Newcastle–Ottawa scale (NOS).

Studies	Year of Publication	Selection	Comparability	Outcome
Redlinger et al. [14]	2013	**	**	*
Liu et al. [15]	2018	**	**	*
Sonesson et al. [16]	2019	**	**	*
Yan et al. [17]	2020	**	**	*
Li et al. [18]	2020	***	**	**
Evans et al. [19]	2021	**	**	*

*, **, ***: NOS evaluates three methodological domains: selection, comparability of cohorts on the design or analysis, and the assessment of outcomes. A star system, with a maximum of nine stars, was used and studies with at least seven stars were considered of high quality.

## Data Availability

Data available upon request to the corresponding author.

## Supplementary Materials

The following supporting information can be downloaded at: https://www.mdpi.com/article/10.3390/jcm12072496/s1, Table S1: P.I.C.O. (patient; intervention; comparison; outcome) model was used to define the clinical questions and clinically relevant evidence in the literature. Table S2: Strategy of the literature research according to the PRISMA statement. Footnote: FTEVAR: fenestrated thoracic endovascular aortic repair.

## References

[1] Czerny M., Schmidli J., Adler S., van den Berg J.C., Bertoglio L., Carrel T., Chiesa R., Clough R.E., Eberle B., Etz C. (2019). Editor’s Choice–Current Options and Recommendations for the Treatment of Thoracic Aortic Pathologies Involving the Aortic Arch: An Expert Consensus Document of the European Association for Cardio-Thoracic Surgery (EACTS) & the European Society for Vascular Surgery (ESVS). Eur. J. Vasc. Endovasc. Surg..

[2] Tsilimparis N., Law Y., Rohlffs F., Spanos K., Debus E.S., Kölbel T. (2020). Fenestrated endovascular repair for diseases involving the aortic arch. J. Vasc. Surg..

[3] Tsilimparis N., Haulon S., Spanos K., Rohlffs F., Heidemann F., Resch T., Dias N., Kölbel T. (2020). Combined fenestrated-branched endovascular repair of the aortic arch and the thoracoabdominal aorta. J. Vasc. Surg..

[4] Mougin J., Charbonneau P., Guihaire J., Schwein A., Tyrrell M.R., Maurel B., Fabre D., Haulon S. (2020). Endovascular management of chronic post-dissection aneurysms of the aortic arch. J. Cardiovasc. Surg..

[5] Spear R., Hertault A., Van Calster K., Settembre N., Delloye M., Azzaoui R., Sobocinski J., Fabre D., Tyrrell M., Haulon S. (2018). Complex endovascular repair of postdissection arch and thoracoabdominal aneurysms. J. Vasc. Surg..

[6] Chastant R., Belarbi A., Ozdemir B.A., Alric P., Gandet T., Canaud L. (2022). Homemade fenestrated physician-modified stent grafts for arch aortic degenerative aneurysms. J. Vasc. Surg..

[7] Bosiers M.J., Donas K.P., Mangialardi N., Torsello G., Riambau V., Criado F.J., Veith F.J., Ronchey S., Fazzini S., Lachat M. (2016). European Multicenter Registry for the Performance of the Chimney/Snorkel Technique in the Treatment of Aortic Arch Pathologic Conditions. Ann. Thorac. Surg..

[8] Li Y., He C., Chen X., Yao J., Zhang T., Zhang H. (2021). Endovascular In Situ Fenestration Technique of Aortic Arch Pathology: A Systematic Review and Meta-Analysis. Ann. Vasc. Surg..

[9] Ahanchi S.S., Almaroof B., Stout C.L., Panneton J.M. (2012). In situ laser fenestration for revascularization of the left subclavian artery during emergent thoracic endovascular aortic repair. J. Endovasc. Ther..

[10] Zhao Z., Qin J., Yin M., Liu G., Liu X., Ye K., Wang R., Shi H., Li W., Jiang M. (2020). In Situ Laser Stent Graft Fenestration of the Left Subclavian Artery during Thoracic Endovascular Repair of Type B Aortic Dissection with Limited Proximal Landing Zones: 5-Year Outcomes. J. Vasc. Interv. Radiol..

[11] Page M.J., McKenzie J.E., Bossuyt P.M., Boutron I., Hoffmann T.C., Mulrow C.D., Shamseer L., Tetzlaff J.M., Akl E.A., Brennan S.E. (2021). The PRISMA 2020 Statement: An Updated Guideline for Reporting Systematic Reviews. BMJ.

[12] Richardson W.S., Wilson M.C., Nishikawa J., Hayward R.S. (1995). The well-built clinical question: A key to evidence-based decisions. ACP J. Club.

[13] Wells G., Shea B., O’Connell D., Peterson J., Welch V., Losos M., Tugwell P. (2013). The Newcastle-Ottawa Scale (NOS) for Assessing the Quality of Non-Randomised Studies in Meta-Analyses. www.ohri.ca/programs/clinical_epidemiology/oxford.asp.

[14] Redlinger R.E., Ahanchi S.S., Panneton J.M. (2013). In situ laser fenestration during emergent thoracic endovascular aortic repair is an effective method for left subclavian artery revascularization. J. Vasc. Surg..

[15] Liu G., Qin J., Cui C., Zhao Z., Ye K., Shi H., Liu X., Yin M., Yang G., Huang S. (2018). Endovascular repair of aortic arch intramural hematoma and penetrating ulcers with 810 nm in situ laser-assisted fenestration: Preliminary results of a single-center. Lasers Surg. Med..

[16] Sonesson B., Dias N., Abdulrasak M., Resch T. (2019). Midterm results of laser generated in situ fenestration of the left subclavian artery during thoracic endovascular aneurysm repair. J. Vasc. Surg..

[17] Yan D., Shi H., Qin J., Zhao Z., Yin M., Liu X., Ye K., Liu G., Li W., Lu X. (2020). Outcomes of emergency in situ laser fenestration-assisted thoracic endovascular aortic repair in patients with acute Stanford type A aortic dissection unfit for open surgery. J. Vasc. Surg..

[18] Li C., Xu P., Hua Z., Jiao Z., Cao H., Liu S., Zhang W.W., Li Z. (2020). Early and midterm outcomes of in situ laser fenestration during thoracic endovascular aortic repair for acute and subacute aortic arch diseases and analysis of its complications. J. Vasc. Surg..

[19] Evans E., Veeraswamy R., Zeigler S., Wooster M. (2021). Laser in situ Fenestration in Thoracic Endovascular Aortic Repair: A Single-Center Analysis. Ann. Vasc. Surg..

[20] Glorion M., Coscas R., McWilliams R., Javerliat I., Goëau-Brissonniere O., Coggia M. (2016). A Comprehensive Review of In Situ Fenestration of Aortic Endografts. Eur. J. Vasc. Endovasc. Surg..

[21] Crawford S.A., Sanford R.M., Forbes T.L., Amon C.H., Doyle M.G. (2016). Clinical outcomes and material properties of in situ fenestration of endovascular stent grafts. J. Vasc. Surg..

[22] Jayet J., Heim F., Coggia M., Chakfe N., Coscas R. (2018). An Experimental Study of Laser in situ Fenestration of Current Aortic Endografts. Eur. J. Vasc. Endovasc. Surg..

[23] Nana P., Spanos K., Dakis K., Giannoukas A., Kölbel T., Haulon S. (2022). Systematic Review on Customized and Non-customized Device Techniques for the Endovascular Repair of the Aortic Arch. J. Endovasc. Ther..

[24] Nana P., Tyrrell M.R., Guihaire J., Le Houérou T., Gaudin A., Fabre D., Haulon S. (2022). A Review: Single and MultiBranch Devices for the Treatment of Aortic Arch Pathologies with Proximal Sealing in Ishimaru Zone. Ann. Vasc. Surg..

[25] Trimarchi S., Nienaber C.A., Rampoldi V., Myrmel T., Suzuki T., Bossone E., Tolva V., Deeb M.G., Upchurch G.R., Cooper J.V. (2006). Role and results of surgery in acute type B aortic dissection: Insights from the International Registry of Acute Aortic Dissection (IRAD). Circulation.

[26] Matsumura J.S., Melissano G., Cambria R.P., Dake M.D., Mehta S., Svensson L.G., Moore R.D. (2014). Five-year results of thoracic endovascular aortic repair with the Zenith TX2. J. Vasc. Surg..

[27] Haulon S., Greenberg R.K., Spear R., Eagleton M., Abraham C., Lioupis C., Verhoeven E., Ivancev K., Kölbel T., Stanley B. (2014). Global experience with an inner branched arch endograft. J. Thorac. Cardiovasc. Surg..

[28] Spear R., Sobocinski J., Settembre N., Tyrrell M., Malikov S., Maurel B., Haulon S. (2016). Early Experience of Endovascular Repair of Post-dissection Aneurysms Involving the Thoraco-abdominal Aorta and the Arch. Eur. J. Vasc. Endovasc. Surg..

[29] Makaloski V., Tsilimparis N., Rohlffs F., Heidemann F., Debus E.S., Kölbel T. (2018). Endovascular total arch replacement techniques and early results. Ann. Cardiothorac. Surg..

[30] Tish S., Chase J.-A., Scoville C., Vogel T.R., Cheung S., Bath J. (2023). A Systematic Review of Contemporary Outcomes from Aortic Arch In Situ Laser Fenestration during Thoracic Endovascular Aortic Repair. Ann. Vasc. Surg..

[31] Eleshra A., Heo W., Lee K.-H., Kim T.-H., Sim S.A., Sharafeldin H., Song S.-W. (2022). Mid-term outcomes of hybrid debranching endovascular aortic arch repair in landing zones 0–2. Vascular.

[32] Qin J., Wu X., Li W., Ye K., Yin M., Liu G., Cui C., Zhao Z., Liu X., Lu X. (2021). Laser fenestration of aortic arch stent grafts for endovascular treatment of retrograde type A dissection. Int. J. Cardiol..

[33] Qin J., Zhao Z., Liu G., Ye K., Yin M., Cui C., Shi H., Peng Z., Jiang M., Liu X. (2019). In situ diode laser fenestration of aortic arch stent grafts during thoracic endovascular aortic repair of Stanford type A aortic dissection. Eurointervention.

[34] Harris K.M., Nienaber C.A., Peterson M.D., Woznicki E.M., Braverman A.C., Trimarchi S., Myrmel T., Pyeritz R., Hutchison S., Strauss C. (2022). Early Mortality in Type A Acute Aortic Dissection: Insights From the International Registry of Acute Aortic Dis-section. JAMA Cardiol..

[35] McWilliams R.G., Murphy M., Hartley D., Lawrence-Brown M.M.D., Harris P.L. (2004). In situ stent-graft fenestration to preserve the left subclavian artery. J. Endovasc. Ther..

[36] Murphy E.H., Dimaio J.M., Dean W., Jessen M.E., Arko F.R. (2009). Endovascular Repair of Acute Traumatic Thoracic Aortic Transection With Laser-Assisted In-Situ Fenestration of a Stent-Graft Covering the Left Subclavian Artery. J. Endovasc. Ther..

[37] Pernot M., D’Ostrevy N., Piperata A., Busuttil O., Labrousse L. (2022). Percutaneous Total Aortic Arch Repair With In Situ Laser Fenestration. Ann. Thorac. Surg..

