# Supplementary material for: Systematic Review on In Situ Laser Fenestrated Repair for the Endovascular Management of Aortic Arch Pathologies
Source: J Clin Med. 2023 Mar 25;12(7):2496. doi: 10.3390/jcm12072496 (PMC10095564; doi:10.3390/jcm12072496)
Supplement: Supplementary file 1 [file jcm-12-02496-s001.zip › jcm-2216841-supplementary.pdf]

Supplementary Tables

Table S1. P.I.C.O. (patient; intervention; comparison; outcome) model was used to define the clinical questions and clinically relevant evidence in the literature.

**P**	Patient, population or problem	Patients with aortic arch pathologies
**I**	Intervention, prognostic factor or exposure	In situ laser fenestrated thoracic endovascular repair (in situ laser FTEVAR) for aortic arch disease
**C**	Comparison of intervention	No comparison performed
**O**	Outcome you would like to measure or achieve	Technical success, stroke and mortality at 30-days and mortality and need for re-intervention during the available follow-up
	What type of question are you asking?	Is in situ laser FTEVAR feasible in patients with aortic arch pathologies?
		Is in situ laser FTEVAR safe in patients with aortic arch pathologies?
		Is in situ laser FTEVAR effective in patients with aortic arch pathologies?
	Type of study you want to find	Observational studies and case series on patients managed in situ laser FTEVAR for aortic arch pathologies

Table S2. Strategy of literature research according to PRISMA statement. *Footnotes:* FTEVAR: fenestrated thoracic endovascular aortic repair.

Frame	Mesh Terms	Search	Inclusion criteria	Exclusion criteria	Sources
P (patients, participants, population)	# 1. "Aortic arch"	#1 AND #2 AND #3 AND #4 AND #5 OR #6 OR #7	Observational studies and case series, reporting on technical success, stroke, mortality and re-intervention of in situ laser FTEVAR<br>Peer-review journals<br>English language	Irrelevant title<br>Irrelevant full text<br>Non-English<br>Editorial, reviews, meta-analyses, technical notes<br>Studies reporting on patients treated with other endovascular techniques, open or hybrid repair<br>Studies reporting on in situ mechanical FTEVAR	Databases (Medline, EMBASE and Cochrane library)
I (intervention)	#2. #3. #4. "in situ" AND "laser" AND "fenestrated"				
C (reference test)	NA				
O (outcome)	#5. #6. #7. "technical success" OR "mortality" OR " stroke" OR "re-intervention"				
Time	Search period: 2000- 2022<br>Last search: 15 October 2022				
